# Gene switching rate determines response to extrinsic perturbations in the self-activation transcriptional network motif

**DOI:** 10.1038/srep26980

**Published:** 2016-06-03

**Authors:** Sebastiano de Franciscis, Giulio Caravagna, Giancarlo Mauri, Alberto d’Onofrio

**Affiliations:** 1 European Institute of Oncology, Department of Experimental Oncology, Milano, Italy; 2 Università degli Studi di Milano-Bicocca, Dipartimento di Informatica, Sistemistica e Comunicazione, Milano, Italy; 3 School of Informatics, University of Edinburgh, Edinburgh, UK; 4 International Prevention Research Institute, Lyon, France

## Abstract

Gene switching dynamics is a major source of randomness in genetic networks, also in the case of large concentrations of the transcription factors. In this work, we consider a common network motif - the positive feedback of a transcription factor on its own synthesis - and assess its response to extrinsic noises perturbing gene deactivation in a variety of settings where the network might operate. These settings are representative of distinct cellular types, abundance of transcription factors and ratio between gene switching and protein synthesis rates. By investigating noise-induced transitions among the different network operative states, our results suggest that gene switching rates are key parameters to shape network response to external perturbations, and that such response depends on the particular biological setting, i.e. the characteristic time scales and protein abundance. These results might have implications on our understanding of irreversible transitions for noise-related phenomena such as cellular differentiation. In addition these evidences suggest to adopt the appropriate mathematical model of the network in order to analyze the system consistently to the reference biological setting.

Since the pioneering investigations by Ko^1^, Kepler and Elston^2^, Lipniacki and coworkers^3^, Karmakar and Bose^4^,^5^, and Mantzaris^6^ the fundamental role of *stochastic gene activation* and *deactivation* rates in driving *transcriptional processes*^7^,^8^,^9^,^10^ and, as recently stressed, determining drug pharmacodynamics^11^,^12^ is acknowledged. In this work, we investigate the relation between the statistical fluctuations of deactivation factors and other signals, and their effect on the dynamics of a basic network motif: the positive feedback of a transcription factor on its own production. Unknown factors and signals are hereby modeled via *realistic extrinsic noises* acting on the gene deactivation mechanism of the network, whose activity is investigated in settings representative of different *cellular types*, *gene switching rates*, *protein amounts*, and *noise properties*.

However simple (and at large extent idealised) it may be, yet this motif accounts for some features essential to orchestrate complex heterogeneous dynamics: *nonlinearity*^13^, *multi-stability*^14^,^15^,^16^,^17^,^18^,^19^,^20^,^21^, *feedbacks*^22^,^23^ and intrinsic and extrinsic forms of *stochasticity*^24^,^25^,^26^. In addition, various settings of network’s functioning are considered by considering *multiple scales* for (*i*) gene switching rates (slow vs. fast time-scale), (*ii*) molecular counts (few vs. abundant copies of the transcription factor) and (*iii*) time-scales of external perturbations (small versus large autocorrelation times of the extrinsic noise). To identify the velocity and temporal scales of, respectively, gene switching and noise dynamics, we will adimensionalize the time by assuming average degradation time of the protein is the new time unit. With small noise autocorrelation time (NAT) we mean a time that is comparable with one; with large NAT we mean an autocorrelation time that is much larger than one. Moreover, we define a suitable scale parameter that tunes the switching rates. Thus, fast switching means that the scale parameter is much larger than one (in the ideal case it tends to infinity), whereas slow switching means that the scale parameter is comparable to one.

These scenarios are representative of specific biological systems; for instance this network was observed to operate in the transcriptional regulation in the yeast GAL1 promoter^27^, in galactose GAL3 signalling switch in yeast cells^28^,^29^, in stochastic mRNA synthesis in mammalian cells^30^ and in human marrow stromal cells differentiation, in response to BMP2 protein stimulation^16^. Also, abundance of transcription factors might be an important determinant of their regulatory activities for a certain function^31^. In the case of few copies copies of such factors, we also consider the network to operate within different volumes, mimicking bacteria versus eukaryotic nucleus systems. Similarly, gene switching rates might be related to trade-offs in metabolic cost associated to network functioning and noise in gene expression, as surrogated by the evidence that for any metabolic cost there is an optimal trade-off between noise and processing speed, which increases for higher cost^32^,^33^,^34^. Finally, extrinsic perturbations with different speeds model variable environmental situations for network activity, such as competing proteins inhibiting or enhancing the natural network feedback via oscillatory synthesis^17^.

As one might expect, the network’s functioning is intimately related to the complex interplay of its scales, suggesting that we should always account for the motif’s operational setting. Our analysis also finds gene switching to shape its dynamics, consistently with earlier observations on the emergence of stochastic fluctuations, sometimes very large, related to that parameter^2^,^9^. This phenomenon drives stochasticity in many genetic network motifs, and by its interplay with nonlinearity it might induce new emergent behaviours^2^,^3^,^7^,^8^,^9^,^10^. For modelers, these evidences should suggest a paradigm-shift to adopt the appropriate mathematical representation of the network consistently to the reference biological setting.

Our work stresses the fundamental role of stochasticity for this type of network, consistently with experimental observations of *stochastic bursts* of RNA production^35^, and randomness induced by transcription factors, proteins or mRNAs present in few copies^36^,^37^,^38^,^39^. Stochasticity can be both intrinsic and extrinsic to a network, and its synergistic effects should be considered to best exploit the predictive power of a model^26^. In fact, models accounting solely for *intrinsic stochasticity* in, e.g., gene on-off switching, are limited to forecast protein bursts. We believe that a crucial step towards a better understanding of regulatory networks requires to account for their interplay with other intracellular networks and random signals coming from the extracellular word. This, in practice, corresponds to a open-world interpretation of a phenomenon and can be achieved by including *extrinsic* forms of stochasticity in the model.

Modelling extrinsic perturbations and their effects is still matter of debate^40^,^41^, although it is clear that the biological scenario goes well beyond the existence of mono-stable noise-reducing networks^42^,^43^,^44^,^45^,^46^. Indeed, since the seventies Prigogine^47^, Haken^48^ and others^49^,^50^,^51^,^52^ have stressed that stochastic–sometimes even deterministic^50^–biochemical systems that are homogeneous in space can exhibit first and second order *“phase transitions*”^42^, that are analogous to the classical “true” phase transitions observed in spatially extended systems^53^. In particular, Horsthemke and Lefever introduced the concept of “noise-induced-transitions” by observing that Gaussian extrinsic noises may allow nonlinear systems to reach multimodal equilibrium states, contradicting previous beliefs^52^. In the framework of bio-molecular networks this means that concentrations of chemicals in response to perturbations may fluctuate around multiple basins of attraction which can lead to novel equilibria^43^,^44^.

The biological potential of exploiting both internal and external noise sources to fluctuate around non-equilibrium configurations and switch among equilibria seem necessary for the emergence of *functional heterogeneity* in changing environments^24^,^54^,^55^,^56^,^57^. For example, phenotype variability in cellular populations, coordination of gene expression across large regulons and, at a longer timescales, evolutionary transitions are probably the most important macroscopic effects of noise-induced phenomena^58^.

## Related works

Investigations on the joint intrinsic/extrinsic stochastic effects for our reference network are dawning. The study by Swain and coworkers is a first attempt in this respect and (*i*) outlined the role of noise autocorrelation, and (*ii*) has stressed that–in this setting–classical white/coloured noises might induce biological artifacts^59^. Many previous works have indeed considered “white” and “coloured” extrinsic *unbounded* noises (i.e., without or with temporal correlation, see Supplementary Material). From a *statistical physics* perspective, it is nowadays accepted that in this and other settings, noises should preserve the positivity and boundedness of the parameters and systems they perturb^25^,^60^. For this reason, this work is mathematically grounded in a recent theory of intrinsically stochastic non-linear networks and extrinsic “bounded noises”^61^,^62^.

For each setting that we consider, we adopt different modeling strategies to describe the network (see Methods). A mean-field approach is appropriate for large number of proteins and absence of extrinsic influences, i.e. mimicking an eukaryotic cell with stable network’s environment. For more complex scenarios, we will switch to adopt approaches from the theory of hybrid Markov processes. The former scenario has been previously studied by other authors^22^,^63^. Consistently with our approach is, for instance, the work by Smolen, Baxter and Byrne^64^ which was used to investigate the differentiation of WB15-M cells in response to BMP2 stimulation in^16^. Interestingly, bimodal behaviour of cellular differentiation observed experimentally was reproduced with impulsive random changes of the transcription factor level: such phenomena emerges for certain settings of our network. Follow-up works made explicit the presence of various forms of intrinsic noise in this circuit^55^,^65^ as well, and allowed to observe its effect for cellular differentiation processes and lactose dynamics^28^. In the framework of continuous approximation of protein concentration (i.e., for abundant copies of the transcription factor), effects of certain extrinsic unbounded noises on protein production have been studied as well^66^,^67^. Also, the interplay between intrinsic and extrinsic unbounded noise affecting protein production in a self-transcription network with sharp and smooth positive feedback has been considered by Assaf *et al.*^68^. For the sake of comparison, and to outline the clear differences between these works and our approach, we provide a detailed commentary of this literature in §3.5 of the Supplementary Material.

## Results

**Figure 1:**
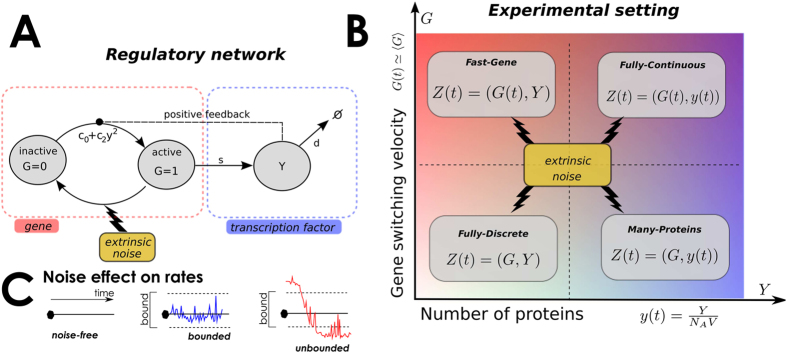
The transcriptional network and the modelling setting. (**A**) the network consists of a gene switching from active/inactive states, with a transcription factor acting with positive feedback on the activation. An extrinsic noise abstractly models the possible unknown interactions of the network with its environment. For instance, if noise has an oscillatory effect on the deactivation rate, its role is to model a certain - potentially unknown - protein synthesised in an oscillatory regime, which competes with the network protein making its deactivation rate oscillate. (**B**) We model the network under four realistic settings where it is observed to operate^16^,^27^,^28^,^29^,^30^, according to the gene on/off switching rate and the number of protein involved. When the gene switching is slow we also test variable volumetric settings mimicking different cell types hosting the network. For any of these settings we use the most suitable mathematical representation of the network, whose robustness is investigated under the effect of bounded extrinsic noises with different features. (**C**) The effect of random noise on the transition rates is that of making them fluctuate as a function of time. In noise-free system rates are constant in time, still being a function of the state values. For noisy system this is no more true, as noise affects the jump times of the process randomly. The difference between bounded and unbounded noise is in the interval spanned by such oscillations. The speed and amplitude of such variations depends on noise parameters.

**Figure 2:**
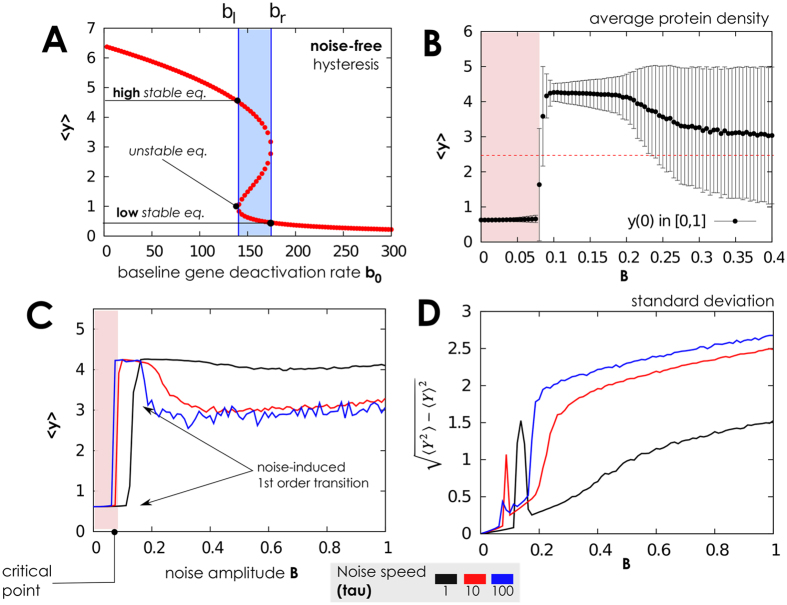
Phase diagrams when the gene switching is fast and many proteins are present. (**A**) Hysteresis plot: bifurcation diagram of the protein equilibrium density against the gene deactivation rate *b*_0_ in this model when noise is absent (*B* = 0). Here we set *s* = 3.2, 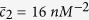 and 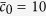. Note that an interval where the system is bistable for 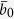 can be easily detected (approx. between the left/right values 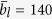 and 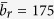). (**B**) Network dynamics perturbed by a Sine-Wiener noise with autocorrelation *τ* = 10. Each points correspond to a simulation realized with initial protein level, *y*(0), randomly sampled in the basin of attraction for the low-level equilibrium, i.e., *y*(0) ∈ [0, 1]. The first-order transition between low, 〈*y*〉 < 1, and high, 〈*y*〉 > 3, equilibrium values for proteins is obtained for very low noise intensity (*B* ≈ 0.066), corresponding to *b*_0_(*t*) close to the lower bound of the hysteresis curve. (**C**,**D**) Different noise autocorrelation times, *τ* = 1, 10, 100, affect the average number of proteins and their variance in a different way. In both diagrams we have set 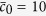.

Our model is pictured in Fig. 1, and its mathematical definition is in Table 1. However, to make more general our results, we scaled time to be dimensionless by taking as time-unit 1/*d*, i.e. by setting *d* = 1. In that table, in line with^5^,^9^, we defined a scale parameter *h* so that the key gene switching-related parameters are written as follows (*c*_0_, *c*_2_, *b*_0_) = 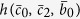. The scale utility parameter is set to *h* = 1 for modeling slow gene switching and *h* → ∞ for fast gene switching . We parametrize the model by converting values previously suggested by Smolen, Baxter and Byrne^64^ and reported in Table 2. For our model we obtain non-dimensional values: *s* = 3.2, *c*_2_ = 1.6*c*_0_*nM*^−2^ and *b*_0_ = 15*c*_0_. By setting 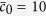 (which implies 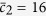 and 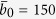) one can easily verify that, in absence of perturbations, the network operating with many proteins and quick gene switching (see Methods) is in its multi-stability region^64^. Namely, the unperturbed system has two stable equilibria at *y*_*L*_ = 0.6268, *y*_*H*_ = 4.28 and one unstable equilibrium at *y*_*U*_ = 1.489. This is further clarified by visualizing 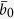 as a bifurcation variable in the hysteresis plot of Fig. 2, where bistability is observed in the (approximate) interval 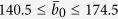. To identify each parameter value to use in this region we performed analytical investigations of this model for generic extrinsic noises. Also, we have performed sensitivity analysis to show that our conclusions are qualitatively invariant to other values of protein transcription rate (see §3.2, Supplementary Material). Finally note that in the scenarios with slow gene switching (where *h* = 1) to 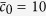 it corresponds a value (*c*_0_ = 10) for the baseline activation rate that is in line with those adopted in the reference work by Jaruszewicz, Zuk and Lipniacki^9^.

Concerning noise, we test two approaches representative of phenomena with different “shapes” (bell versus horn, Supplementary Figure S1 and §1.1) which are called *sine-Wiener* and *Cai-Lin* noises^69^,^70^,^71^,^72^. In detail, these noises are discussed in §1.1. of the Supplementary Material; their amplitude, *B*, and autocorrelation time, *τ* (which can be considered as the characteristic time of a colored, bounded or not, noise^73^), are here ranged over plausible values to span different network’s operational settings. We stress that, in the settings that we consider for this model, canonical unbounded approaches lack to re-produce the forecasts that we obtained with these bounded noise–hence introducing the problem of distinguishing modeling artifacts from realistic predictions (Supplementary Figure S4 and §3.6)–and that the introduction of noise acting onto the activation rate does not allow different forecasts, but rather nullifies extrinsic effects (Supplementary Figure S2 and §3.3). Also, we remark that our results might change, quantitatively, according to the parametrization of the model and the type of boudned noise adopted. However, the qualitative nature of our predictions shall be consistent with a wide range of possible parametrizations.

By simulation ensembles, we provide an empirical estimation of the *stationary probability density* of the number of proteins (or their concentration, if more appropriate), as well as of derived summary statistics for their average and standard deviation. To avoid biases by analysing transient behaviour, we estimate these statistics in the long run by simulating the network activity in the interval [0, 10^4^].

### Network operating with large amount of proteins and fast gene switching

An analytical study of the *transient probability density* of the number of proteins (§3.1, Supplementary Material) allows preliminary forecasts. This is the only case in which we were able to carry out an analytical study of the network dynamics, which we then validated via simulations.

We can indeed observe that, in this scenario, the network resists to extrinsic perturbations if these have small (but not infinitesimal) amplitude *B*. Namely, in such a case we showed that the system has two stationary attractors. As a consequence, the number of proteins is consistent with the basin of attraction in which the network starts functioning, for it being attractive towards the low or high equilibria. The more the strength of the noise increases, the more its effect on the network can be appreciated, mimicking a network response to perturbation. For intermediate and constant *B* an irreversible switch among equilibria (low to high, or viceversa) is observed. The higher gets *B* the less “irreversible” becomes such a transition, with the final forecast of the network oscillating among equilibria for large *B*. This suggests that the network can resist only to certain perturbation intensities, since *first and second order phase transitions* (as meant by^42^,^47^,^48^,^49^,^50^,^51^,^52^,^73^) emerge as noise increases^53^,^50^. The first-order transition encompasses a sudden increase of the average number of proteins, 〈*y*〉, the latter its smooth decrease; in both cases, in the transition point, a widening of the standard deviation for *y* is observed. Note that the observed first order *phase transition* corresponds to the sudden passage of the system from a scenario with two distinct stochastic attractors to a scenario with one attractor (and vice-versa), as in classical phase transitions^74^,^75^.

Numerical simulations are consistent with these predictions in Fig. 2, where the statistical aggregates for *y* are plot against noise parameters, for both sine-Wiener and Cai-Lin noises. In there, the transitions are found for two possible values of *B* (approx. 0.066 and 0.166). For noise intensity below the first threshold the equilibrium distribution is unimodal and peaked on the protein level corresponding to the initial protein count. Thus, the basins of attraction for high and low protein levels are separated by unstable fixed points. The first-order transition is also well characterised by the divergent variance around the transition point. Interestingly, the speed of noise’s variation determines where these phase-transitions are predicted. In particular, for autocorrelation greater than a certain threshold–here, empirically estimated to be 10 by comparing *τ* = 10 and *τ* = 100 in Fig. 2–the quantitative differences between predictions diminish. Nonetheless, there is a clear quantitative difference between predictions of values of 〈*y*〉 for *τ* = 1 and *τ* > 10; the *qualitative* trend is instead consistent across all the values of *τ* that we report in the figure. All in all, for a very quick perturbation regions get smaller while the variance of *y* significantly increases. This confirms that the second-order transition is characterised by stochastic oscillations with amplitude increasing with *τ*. Quite interestingly, these predictions are rather similar for both the types of noise that we considered (data not shown).

**Figure 3:**
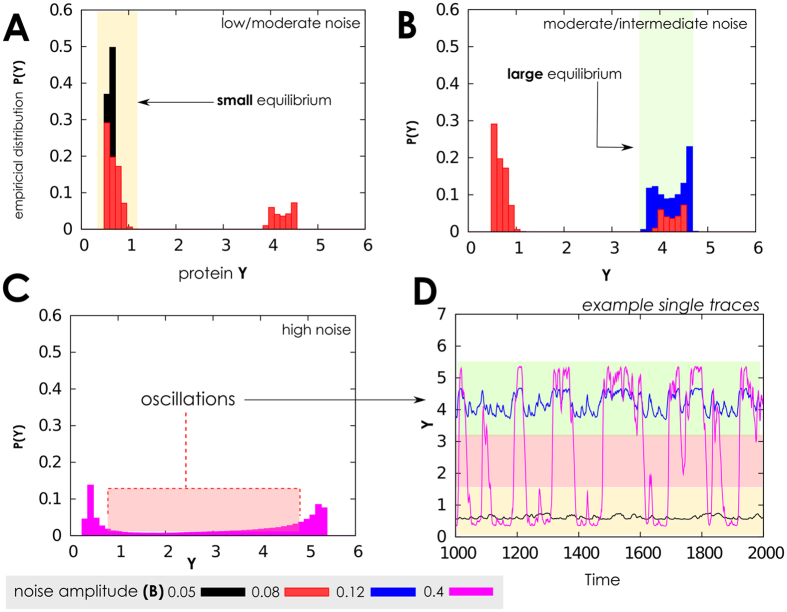
Protein distribution when the gene switching is fast and many proteins are present. (**A**–**C**) Protein probability densities *P*(·) obtained when the network operates in the same setting of Fig. 2. Here we use different values for noise amplitude, *B*, to mimic the different intensity with which noise might interfere with the baseline gene deactivation rate. The first-order transition between low and up protein levels emerges with low noise intensity (*B* ≈ 0.08), while a second-order one, between up level and oscillating up/low levels emerges for higher values, consistently with what is observed in Fig. 2 (parameter values set in the same way). (**D**) Example time-series of network dynamics for 1000 time units is shown with different values of *B*. Observe that for certain noise intensities (e.g., *B* = 0.4) the protein level oscillates consistently between its two equilibrium states.

The effect of the noise amplitude on the stationary probability of proteins, along with some simulated time series, are shown in Fig. 3. As expected, for small noise amplitude proteins fluctuate around small values which gets higher for higher noise amplitude. Finally, for larger *B* a bimodal density is observed corresponding, in the time series, to oscillations between large and small protein amounts. These results could shed new lights on the relation between the strength of these interactions, the surrounding environment and the amount of produced proteins. In this specific context, we could speculate on the presence of a certain inhibitor, which either directly or indirectly inhibits RNA polymerase from transcribing protein *y*–thus silencing the activity of gene *G*. This inhibitor is produced by a certain network that we are not willing–or that we could not–model in this context. In this case, hence, *B* can be interpreted as the “magnitude of fluctuations in the density of this unknown inhibitor”, and *τ* (noise autocorrelation) can be interpreted as a (function of) the characteristics production and degradation times of the inhibitor.

### Network operating with few proteins and slow gene switching

We compare forecasts of protein dynamics obtained when the network operates in this setting against those obtained in the opposite case. In this case, we set a normalization constant to retrieve protein density as of a reasonable order of magnitude of protein numbers involved. To be realistic, since bacteria’s volume and eukaryotic cellular nucleus oscillate in the volumetric range of 10:10^3^ *μm*^3^, we explore values in 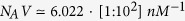 76,77. Moreover, to efficiently calculate phase diagrams for this model we approximated an exact algorithm by observing that the network and noise time-scales are very well separated^61^. To check the consistency of this approximation, the probability density of protein counts is double-checked with the one predicted by the exact algorithm.

**Figure 4:**
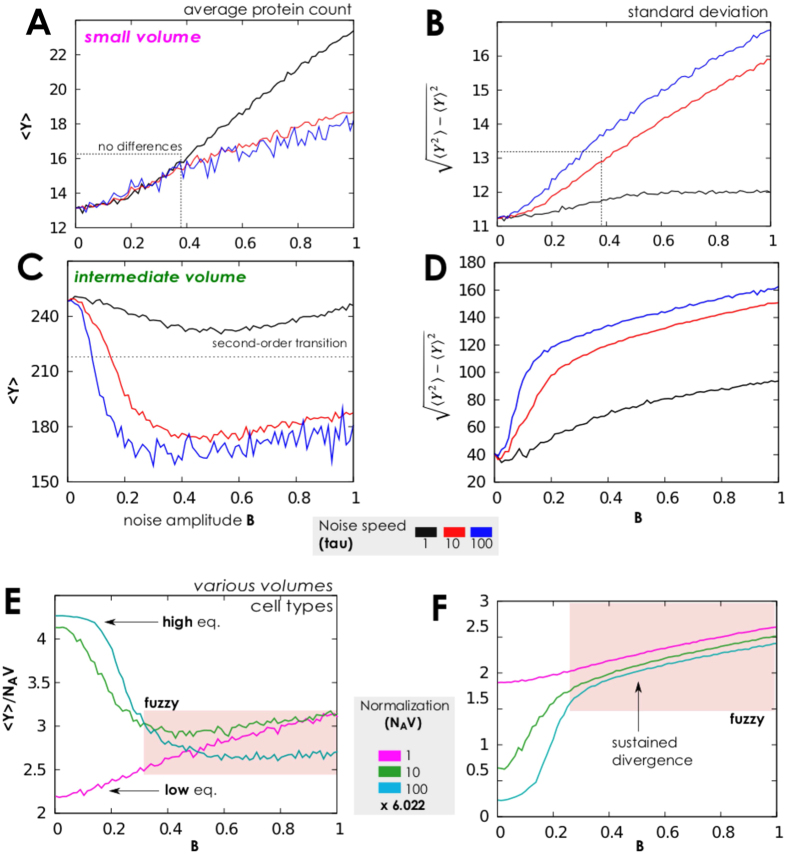
Phase diagrams when the gene switching is slow and few proteins are present. (**A**,**B**) Average number of proteins and standard deviation of protein density, when the network is perturbed by a Cai-Lin noise (*z* = −0.5) with different speed of variation, *τ* = 1, 10, 100, low normalisation (*N*_*A*_*V* = 6.022, mimicking a small cellular volume), and 1000 initial proteins. The typical state is oscillating between high and low protein levels, and for low *τ* the protein equilibrium states become “fuzzy“, up to a point that it is not possible to distinguish among equilibria (see also Fig. 6). (**C,D**) We depict the same situation of panels *a* and *b* but with a tenfold volumetric value, and initial condition randomly sampled in *Y*(0) ∈ [0, 100]. This allows to observe the emerge of the second-order transition between high protein level and the oscillating regime as noise intensity increases. (**E,F**) We here show that results shown in previous panels seem irrelevant of the type of considered noise. We indeed use in these figures a sine-Wiener noise, initial condition *Y*(0) ∈ [0, 10 × *N*_*A*_*V*/6.022] and compare the same volumetric settings used in the other panels.

**Figure 5:**
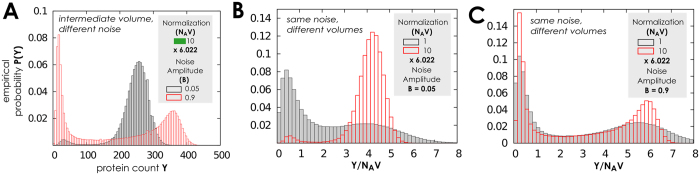
Protein distribution with slow gene switching, few proteins and sine-Wiener noise. (**A**) When the network operates with low gene switching rates, few proteins, and volume is intermediate, *N*_*A*_*V* = 60.022, we still observe the usual second-order transition from high protein level to an oscillating state in response to a sine-Wiener noise (with *τ* = 10). Observe that even with a very small bound value the system can rarely switch off the large protein level, resulting in a small left residual peak in the distribution. (**B,C**) Normalised protein density distributions, 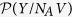, as a function of the available volume, *N*_*A*_*V*, and noise intensity. Noise amplitude *B* enhances the peak corresponding to the low protein level and increase the gaps between equilibria with many or few proteins. In all plots we set as protein initial condition *Y*(0) ∈ [0, 10].

In this setting the network seem to adapt differently to the presence of noise, and seem to behave differently according to volume it has available (see also §3.4, Supplementary Material). Note that our results gain interest as the discretization of a model (i.e., of *Y*) do not necessarily preserve the properties of its continuous counterpart (i.e., of *y*, see Supplementary Figure S3). In our case, in fact, no first-order transitions are observed in this case, and for low noise the predicted equilibrium depends on volume values via normalization; see Fig. 4. For large volumes representative of, e.g., eukaryotic nucleus, oscillations between equilibria are still observed. The protein density distribution and the time series in Fig. 5, reveal that in this operational setting the network residence in the equilibrium with large levels of transcription is very effective for weak noise. In the density distributions this results in a small residual peak for low protein numbers.

For higher volume values, *N*_*A*_*V* ≥ 60, the average value 〈*Y*〉 increases and the second-order transition to an oscillating state is predicted, see Fig. 4 panels (C,D,E,F). For some cases, this has a large ratio between variance and average protein values, *σ*/〈*Y*〉, and small 〈*Y*〉. Thus, the more proteins are present–as of normalization–the more separate and well distinguishable are the up/low protein states. Precisely, the *second-order phase transition* (as meant by^42^,^47^,^48^,^49^,^50^,^51^,^52^,^73^) emerges for sufficiently quick noise, i.e., high *τ* value. Indeed, for *τ* = 1 we can not find reasonable variance in *y*, compared to its mean. However, for *τ* = 10 the average protein count is 180 and the variance is about 120–observe also Fig. 5 for *B* = 0.9. Thus, we can speculate that there exist an optimal value for noise speed to define when the system switches from low to high equilibria of protein counts. For modelers, this confirms that it is crucial to use an exact modelling approach to track the correct number of proteins and make as precise as possible forecasts. The protein density distribution in Fig. 5 illustrates these results; for very small noise amplitude, *B* = 0.05, the network rarely switches off the large level of proteins, the density distribution is “quasi unimodal” and the residual peak at low protein numbers is small.

**Figure 6:**
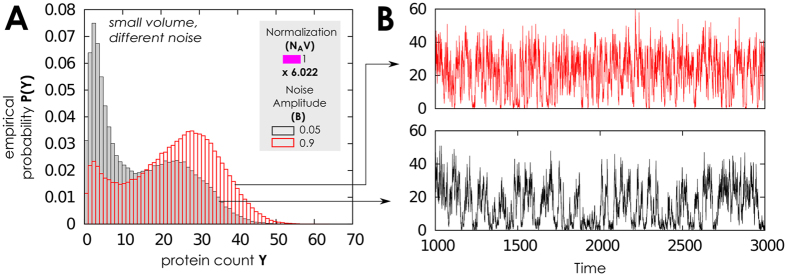
Protein distributions and corresponding time series with slow gene switching and few proteins. (**A**) We test here response to a very slow noise, *τ* = 1, when the network has low gene switching rates and few proteins. Here we consider the case of a small volume available being *N*_*A*_*V* = 6.022 and the protein initial condition *Y*(0) ∈ [0, 6.022]. (**B**) One can observe that in this case *B* enhances the peak corresponding to the high level of proteins. In this case it is also possible to appreciate the intrinsic stochasticity of the small protein numbers; this indeed mixes down the protein states, up to a point in which the two distinguish equlibria can not be clearly separated anymore.

Noise correlation time, jointly with amplitude, contributes to shaping these probability densities as well. For *τ* ≤ 10 amplitude enhances the peak corresponding to the low protein level and increase the high/low protein states gap, see Fig. 5(B,C). On the contrary, for low autocorrelation, *τ* = 1 in Fig. 4(A,B), *B* has the effect to enhance the peak corresponding to the high protein level, see Fig. 6.

### Network operating with few proteins and fast gene switching

**Figure 7:**
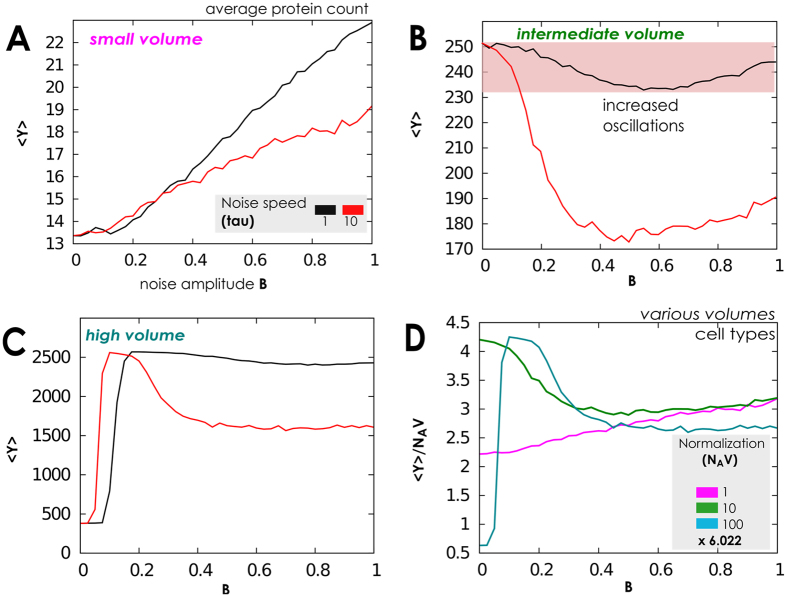
Phase diagrams when the switching rate is fast and few proteins are present. (**A–C**) For a sine-Wiener noise with different autocorrelation values we test network’s response as a function of the available volume by spanning *N*_*A*_*V* over three orders of magnitude. This allows to observe that a low noise autocorrelation *τ* has double effect: it increases the amplitude of the oscillating state, and enlarges parameters space attracting the dynamics to a low equilibrium value for *Y*. (**D**) We test if noise speed plays a role in this phenomena, as a function of volume. Here we use the same noise with *τ* = 10 and show the normalised distribution of proteins 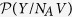. When noise amplitude is small, a re-entrant transition in the normalised mean number of proteins emerges. In all panels we set *Y*(0) = 〈*G*(0)〉 = 0.

**Figure 8:**
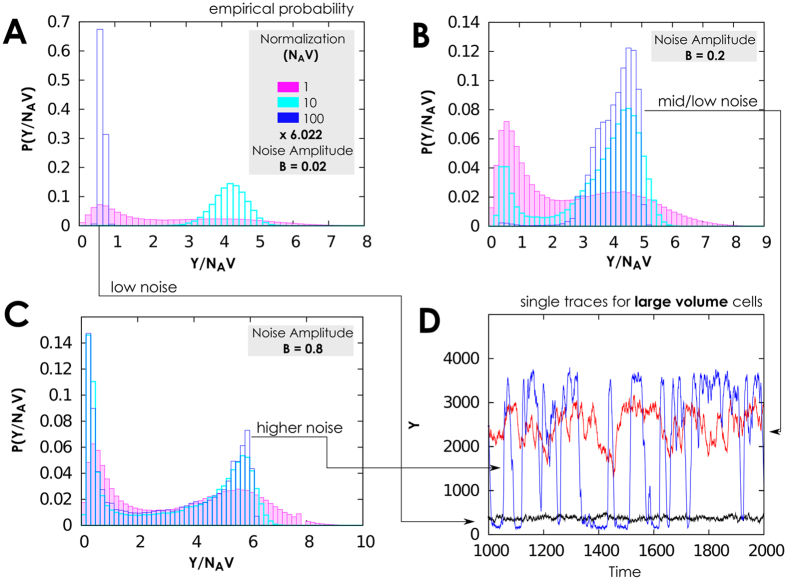
Protein distribution with fast gene switching and few proteins. (**A–C**) In this case we test response to a sine-Wiener noise with different strength when the network operates with different volumetric settings. We test here different noise amplitudes. For *B* = 0.02 there is a re-entrant transition in the normalised protein number, *Y*/*N*_*A*_*V*, and protein density distribution 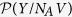 switches from bimodal/oscillating to unimodal/high level, and finally to unimodal/low level. (**D**) Example time series when volume is very large, *N*_*A*_*V* = 602.2. In all panels we set *τ* = 10 and the initial conditions are *Y*(0) = 〈*G*(0)〉 = 0.

In this setting the model behaviour strongly depends on the volumetric setting in which the network operates, *N*_*A*_*V*, and the speed of the extrinsic noise, *τ* (see Supplementary Figure S3). We adopt *B* as parameter to identify three different regimes which are shown in Figs 7 and 8: (*i*) for small volume the system oscillates, (*ii*) for intermediate volume a second-order transition form large to an increasingly oscillating behavior is observed; (*iii*) for sufficiently large volume we recover, as in the case of fast gene-switching and large number of proteins we observe first-order and second-order transitions.

When noise amplitude is small, a re-entrant transition in normalised protein number *Y*/*N*_*A*_*V* emerges, and the normalised protein density distribution switches from bimodal/oscillating to unimodal/high level, and finally to unimodal/low level, see Figs 7(D) and 8(A). Important consequences on cell biochemical equilibrium could be deduced from this phenomenology, in particular regarding cell mitosis, when the cell volume increases and the disaggregation of cell nucleus and the final division in two daughter cells change dynamically the normalisation term. We reserve a deep analysis of the nature of this transition in future works (see Conclusions). Analogously to setting in which the network operates with a large amount of proteins and fast gene switching a lower noise autocorrelation time enhances the low protein region and increases the amplitude of oscillations, as depicted in Fig. 7(B,C). Thus gene-switching deeply influences the network activity in this case.

### Network operating with large amount of proteins and slow gene switching

When the switching times of the genes expressing the self-regulating transcription factor are of the same or lower order than the degradation time of the protein, the effects of the bounded noise are very similar to the ones for slow-switching and few proteins, in the limit of high volume. Thus the first-order transition for fast-switching and many proteins disappears, i.e. for low *B* values the equilibrium corresponds always to many proteins.

**Figure 9:**
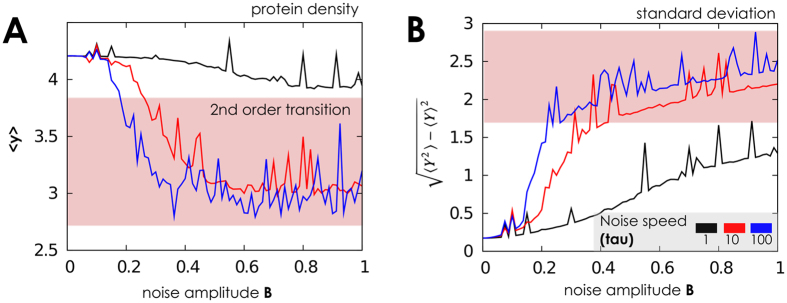
Phase diagrams with slow gene switching and many proteins. (**A,B**) We use here a Cai-Lin noise with *z* = +0.5. Analogously to the second-order transition, predicted for high volume values when few proteins are available, we observe a transition from an high protein level equilibrium, with a unimodal distribution, to an oscillating state, characterised by a bimodal protein distribution. See also Figs 4 and 5.

Analogously to the second-order transition we observe a transition from an unimodal (high protein level equilibrium) to a bimodal protein distribution with both sine-Wiener and Cai-Lin perturbations, in a specific range of noise intensity (*B* ≈ [0.1, 0.3]), see Fig. 9. This transition has the same dependence from the type of noise and its autocorrelation time as is the case of the network operating with large amount of proteins and fast gene switching (compare Figs 2 and 9).

## Discussion

We performed an exhaustive computational analysis of a minimal transcriptional network (i.e., a motif) dynamics and its response to the effects of realistic stochastic perturbations hitting gene deactivation rate. Of course, extrinsic perturbations can virtually act on subprocess constituting the motif in study. In particular, on the activation (§3.3, Supplementary Material) and the degradation rates, where the presence of noise is also proxy of the binding of the TF with other chemicals. Thus, on the one hand the present work is meant as a first step of a more long-range research, where the co-presence of such noises has to be taken into the account, and where mutual correlations between extrinsic noises will very likely play an important role. On the other hand, we believe that our results are of interest since one of key features of theoretical models in biology (and elsewhere) is the capability of disentangling single phenomena, which is much more difficult and often impossible in the wet experiments.

The network functioning was assessed in different experimental settings where it might operate, possibly collecting settings representative of distinct cellular types, abundance of transcription factors and ratio between gene switching and protein synthesis rates^16^,^27^,^28^,^29^,^30^. In all settings it was excluded that the predicitons were trivial or dependent on modeling artifacts; sensitivity analysis was also carried out to show that our conclusions are qualitatively invariant to other values of protein transcription rate. Results suggest that, in general, gene-switching rate is the key parameter to modulate network’s response to external perturbations. In some specific situations, such as when the network operates with a small amount of proteins available, response seem to be largely dependent from cellular volume, which suggests us that the very same network might exhibit different quantitative dynamics when hosted by different organisms, or when operating in different locations of the same organism.

Concerning extrinsic noises, of course, it would be important to get experimental information on the statistical properties of extrinsic noises affecting specific types of cells, and specific transcription networks. In absence of such information, and in a generic cell setting, we tested two different types of perturbations - one with asymptotic bell-shaped distribution, another with horn-alike shape. For both, we tested different strengths and speed of variation in their effect on the network, possibly mirroring different settings of perturbation. Quite surprisingly, results suggest that little can be imputed to their different distributions, while much of response is imputable to how strong noise hits on the gene deactivation rate. In particular, as noise increases a cascade of phase transitions is observed, from first to second-order, with predictions slightly different according to the operational setting for the network. Indeed, these transitions among equilibria - from hight to low and viceversa, with possible situations of irreversibility and persistent oscillations - are clearly observable when the network gene switching is fast and a large number of proteins is available. In this case predictions suggest that once the protein concentration switches from low to high values, or viceversa, there is no backward switch unless noise amplitude is further increased. This type of “irreversible” response seems imputable to gene switching rate, since no fist-order transitions were observed for low rate values. Interestingly, this same dynamics is observed even when few proteins are available provided cellular volume being large. The autocorrelation time characteristic of the extrinsic noise was also studied, mimicking for instance the presence of inhibiting proteins competing for the same transcription sites of the gene, and synthesised at different velocities. Apparently, autocorrelation affects solely the second-order (smooth) transitions by amplifying the probability to observe few proteins, and leading the network to an oscillatory dynamics where proteins span between high and low values periodically.

To obtain these results we applied careful considerations on the physics of this network to adopt suitable mathematical representations of our model. These were based on steady-state hypotheses of either gene or protein dynamics, and required to adopt–and sometimes adapt–state-of-the-art techniques for biophysical simulation of intrinsic/extrinsic stochastic systems. On one side, our results should stress the importance of adopting the appropriate mathematical network’s representation, consistently to the reference biological setting where it operates. When this aspect is underestimated, mathematical forecasts might be artefacts induced by unsuitable modeling approaches, as already noted in previous works^78^.

Besides these evidences representing general caveats for modelers, the biological interpretation of our forecasts seem even more appealing. The noise-induced *irreversible* first-order transitions among protein levels of the transcription factor could be cautiously read as a mechanism, employed by bi-potent cells where the factor is abundant and the gene switching is very fast, for the choice of a *permanent* cellular fate. Indeed, for these cells, as the amount of proteins is high, the fate-choice imputable their intrinsic stochasticity seems irrelevant. Thus, heterogeneity of fates in such a cellular population might originate by the interplay of noisy circuits with a certain minimum strength of influence one another; in this case noise amplitude could be seen as a “static” random variable. Of course, these arguments are quite speculative and they would require further theoretical and experimental investigations to be supported.

Finally, for the case of large number of molecules and fast gene switching, we note that, thanks to the boundedness of the perturbation, for small (but non infinitesimal) amplitude of noise the system (and its associated Fokker Plank equation, of course) exhibits two distinct stochastic attractors (two distinct *phases*), in agreement with the biological intuition. The observed first order *phase transition* corresponds thus to the sudden passage of the system from this scenario to a scenario with one attractor, and *vice-versa*. These phenomena are an hallmark of classical phase transitions^75^ (as discussed in depth in^74^), and are rarely observed in non-spatial systems. This makes the observed first-order transition quite similar to classical phase transitions.

More in general, it is important to stress that the network we study is quite abstract and generic. Of course, a more realistic description of the gene-switching process might lead to other biological predictions of interest. For instance, our model neglects some important macroscopic features such as the explicit presence of mRNAs and the spatial distribution of molecules^57^,^65^. Similarly, accounting for potential delays in the transcription and degradation processes might lead to interesting predictions as well, and could be done at a low effort by employing known simulation techniques for non-Markovian processes^79^,^80^. Also, in this work we focused on parameter settings for which the network dynamics is predicted to be bistable, at least when a large amount of proteins is present and gene switching is quick. Clearly, this was done to highlight the potential emergence of first-order phase-transitions. As a future investigation, however, a more canonical investigation of the parametric regions inducing monostability might be amenable. Such an analysis would allow to exhaustively study the role of noise in determining bimodal protein dynamics. Notice that we can however exclude that adding other sources of noise, in the current formulation, could have led to more interesting predictions, as the extrinsic effects on the network dynamics vanish (Supplementary Figure S2).

All of the above considerations depict a complex scenario, and motivate at least three, more substantial, issues worth further investigations. The first concerns the behaviour of the network in cycling cells: indeed, since in the case of few proteins cellular volume plays a relevant role, it is natural to ask what might happen in cycling cells when this parameter varies in time, due to mitosis. The second issue is related to the dichotomy between physiological and abnormal cells. Here we investigated normal cells where the number of genes, 2, is conserved through time. Abnormal cells, which might have more/less copies of the gene, might allow to observe phase transitions which are hereby unobservable. Along this line, first-order transitions might be observed concomitant with genetic amplifications and a mean-field behaviour of gene-switching could emerge also when this process is slow. A positive answer to this question might lead to further investigations on the correlation of this phenomena with proteins which are over-expressed in cancer cells. As far as cancer as well as physiological cellular populations are concerned, it would be of interest to assess the impact of realistic extrinsic noises affecting single cells *at the population level*, by suitably extending the approach proposed by^6^. Finally, experimental evidences showed that the “on-off” two-states gene switching is often an oversimplification^81^,^82^,^83^. Exploring multi-state and more complex mechanisms of gene dynamics is thus an important further issue. In particular, it is of interest the elegant model of a self-activating transcription factor proposed by Karmakar and Bose^5^, where the binding of the gene to a dimer does not imply the immediate genic activation. The introduction of such mechanisms might lead to an additional variability, similar to those we hypothesized for gene amplification.

## Methods

### A transcriptional network with positive feedback

We consider a simple transcriptional network constituted of a gene *G* and its protein product, a transcription factor with positive feedback on its own gene. We also assume that the deactivation rate of the gene is under the influence of bounded extrinsic perturbations which affect the deactivation rate. The network is pictured in Fig. 1.

The protein numbers will be denoted as *Y*, and its concentration as *y* = *Y*/*N*_*A*_*V*, where *V* is the cell volume and *N*_*A*_ Avogadro’s number. Proteins degrade at rate *d*. In our applications we consider the the transcription factor to be produced by two copies of *G* (diploid case, *n* = 2). However, in pathological cases more/less gene copies might be needed: e.g., due to gene heterozygous loss it may be *n* = 1^84^, while in tumour cells it may be *n* > 2, due to, e.g., poliploidity^85^.

In the above network a single gene copy switches among being either *active* and producing its transcript with rate *s*, or *silent*. Thus, at each time instant we model the generic i-th gene as a binary variable where *G*_*i*_ = 1 and *G*_*i*_ = 0 correspond to the former and the latter situation. Notice that since the transcript protein positively feedbacks just on its own production, it does not act in the process of gene deactivation. Thus at time *t*, the total number of activated genes is: *G*(*t*) = *G*_1_(*t*) + *G*_2_(*t*), with *G*(*t*) ∈ {0, 1, 2}.

Gene activation and deactivation rates are function of the number of protein performing feedback and the intensity of the extrinsic perturbation, respectively. The former is *c*(*y*) = *c*_0_ + *c*_2_*y*^2^ with baseline value *c*_0_ and positive feedback modelled by the additive term *c*_2_*y*^2^. The latter has form


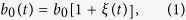


where *b*_0_ is its baseline value and 1 + *ξ*(*t*) is the model of the extrinsic perturbation, which varies in time. Here, *ξ*(*t*) is a *bounded extrinsic noise* varying at most in the interval [−1, 1] and with average value 0. Thus, it fulfils two conditions: the deactivation rate is always positive, *b*_0_(*t*) 0, and its average value is the baseline rate, 〈*b*_0_(*t*)〉 = *b*_0_, ensuring a non-bias situation towards certain values of *b*_0_(*t*)^61^.

Every noise is a random process (extrinsic stochasticity) characterized by (at least) two parameters: an *amplitude* - sometimes called also “intensity” −*B* ∈ [0, 1], and an *autocorrelation time τ* 0. For example, this could be the following *Sine-Wiener* model of noise


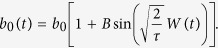


Example of effects of noise on rates is in Fig. 1. Roughly, *B* determines how much *ξ* spans over the interval [−1, 1] by reducing it to [−*B*, *B*], while *τ* sets the noise speed in changing values over time. In the Supplementary Material, §1.1, we provide a detailed description of both the Sine-Wiener and the *Cai Lin* noise that we use in this paper. From a modelling perspective, this is an attempt at considering an abstract representation of the possible unknown interactions of the network with its environment. For instance, when *ξ*(*t*) is randomly fluctuating we are modelling an unknown proteins synthesized in an oscillatory regime, which compete with the transcription factor making its gene deactivation rate stochastically fluctuate. Notice that parameters choice for *ξ*(*t*) should be consistent with its biological interpretation, and that this approach could be extended to test noise effects on all network’s components. However, we restrict our analysis to its effects on deactivation, and postpone further investigations to future works.

### Biological settings of network’s functioning

As mentioned in the Introduction, we want to consider this network as operating in different biological settings according to its characteristics “scales”, pictured in Fig. 1. Four different experimental settings emerg from two, out of the three possible, involved scales, namely: (*i*) *the slow, respectively fast, rate of gene switching among active/inactive states* and (*ii*) *the small, respectively large, number of transcription factors*. Then, in each of those settings we will assess the network response to extrinsic bounded noise with different features. This makes the network a simple - but powerful - *multi-scale* system where both temporal and numerical scales coexist. Some separate contributions in the literature exist which studied this network in specific scenarios: deterministic^63^,^65^, intrinsically stochastic^9^,^65^ and under the effect of unbounded white and coloured Gaussian perturbations^66^,^67^,^68^. We provide a detailed commentary of some of these works in §3.5 of the Supplementary Material; however, to the best of our knowledge, a thorough combination of these experimental settings augmented with extrinsic noises was not studied before.

From a modeling perspective every setting allows us to adopt different mathematical techniques to represent and simulate network dynamics, the point being to investigate if setting-dependent forecasts of different chemical concentrations can be produced. In principle, the Markov processes modeling approach introduced by Gillespie could be implemented in all settings^38^. This would lead to count explicitly the number of proteins and states of the genes in the network (*exact model*), with *scalability issues* emerging from the presence of non-homogenous scales (§1.2, Supplementary Material). Since this approach would turn out to be impractical to carry out exhaustive analyses, we apply careful mathematical considerations on the physics of this network to adopt suitable approximated mathematical representations based on *Steady-State Hypotheses* (SSHs) of either genes or proteins.

Our methodological approach is in line with the recent important analytical work by Jaruszewicz *et al.*^9^ on the impact of unperturbed intrinsic gene switching, with the fundamental exceptions that we include extrinsic noise and more than one gene, and reproducing the analytical investigation of that work in presence of realistic extrinsic noises was unfeasible. The modelling techniques that we employ are, in order of decreasing complexity, chosen according to the paradigm shown in Table 1 and each model instance is shown in Table 2. Further discussions on the derivation of the models are in §2 of the Supplementary Material. In general, these approaches result is a series of models of decreasing complexity both in terms of mathematical representation and cost of simulation, which we now describe.

### Model with slow gene switching and small number of proteins

Here we *account explicitly* for the internal states of each gene, *G*, and the amount of transcribed proteins, *Y*, via a Markov process with discrete state **Z**(*t*) = (*G*, *Y*). Coupled with noise, this is a *time-inhomogeneous birth-death process* because of the effect of noise on the deactivation rate, i.e., *b*_0_(*t*) which contains the noise term *ξ*(*t*). We used the notation *G* → *G* + 1, i.e. a gene switches to activity, to denote a birth event for *G* in the time interval (*t*, *t* + *dt*); similarly, we denoted a death event such as protein degradation with *Y* → *Y* − 1.

This exact network representation can be simulated efficiently only when a few hundreds of proteins are present and the time-scales of the involved events are homogenous; if this is this case, an exact algorithm can be used^25^,^61^. If one sets noise intensity to *B* = 0, then this process becomes time-homogeneous^86^.

### Model with fast gene switching and few proteins

We consider the case of a switching rate of the gene to be fast enough to satisfy 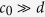 and 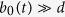, namely baseline activation and deactivation rates being higher than protein degradation. This is equivalent to assume *h* ≫ 1, i.e. in the idealized case *h* → ∞. In this case gene switching is very quick, so we can track just the average number of active genes, *G*(*t*) ≈ 〈*G*(*t*)〉^2^,^9^, which yields


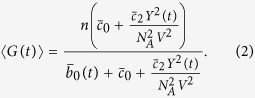


From which follows the equation by considering protein density *y*(*t*). Here the network state is **Z**(*t*) = (*G*(*t*), *Y*) and aggregates the effects of deactivation, activation and feedback events in a unique mean-field approximation of 〈*G*(*t*)〉, a continuous component. Protein counts are tracked explicitly and the resulting process coupled with noise is simulated by extending the technique introduced in^61^ to a stochastic hybrid system, in a natural way.

### Model with slow gene switching and large number of proteins

With low gene-switching rate and large number of molecules, we can replace the protein rate equations with the following mean-field model for *protein density y*(*t*), and introduce a differential equation for 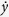 which depends on the number of active genes, *G*. Thus, we are here considering a model state **Z**(*t*) = (*G*, *y*(*t*)), and we are aggregating the effects of transcription and degradation in *y*(*t*), resulting in both deactivation and feedback events to become time-inhomogeneous. This case is symmetrical to **B**, is again a hybrid process and is simulated in the same way.

### Model with fast gene switching and many proteins

A double approximation of *G* and *Y* combines approaches used in **B** and **C**, yielding one differential equation augmented with the equation defining the noise. Here, the equation for protein density binds 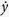 to 〈*G*(*t*)〉; in the noise-free case this results in the well-known ordinary differential equation introduced by Smolen-Baxter-Byrne^64^,^66^,^67^,^68^; a commentary on the differences between this model and our approach is provided in §3.5 of the Supplementary Material.

## Additional Information

**How to cite this article**: de Franciscis, S. *et al.* Gene switching rate determines response to extrinsic perturbations in the self-activation transcriptional network motif. *Sci. Rep.* **6**, 26980; doi: 10.1038/srep26980 (2016).

## Supplementary Material

Supplementary Information

## Figures and Tables

**Table 1:** Modeling paradigm.

Model	Rate†	Proteins‡	*Example biological setting*	SSH	Suitable modeling approach*
**1**	*slow*	*few*	Transcriptional regulation in the yeast GAL1 promoter^27^	–	Markov process for both genes and proteins
**2**	*fast*	*few*	Galactose GAL3 signalling switch in yeast cells^28^,^29^	*G*	A differential equation for genes coupled with a Markov process for proteins
**3**	*slow*	*many*	Stochastic mRNA Synthesis in Mammalian Cells^30^	*Y*	A Markov process for genes coupled with a differential equation for proteins
**4**	*fast*	*many*	human marrow stromal cells (WB15-M) differentiation, in response to BMP2 protein stimulation^16^	*G*, *Y*	Coupled differential equations for both genes and proteins

We consider the network as operating in different scenarios, which are summarized in this table. Every of these settings is obtained once any of the characteristics scales of the network is fixed: these are the number of available transcription factors and the rates of genes switching among active and inactive states. A further scale is represented by cellular volume, and applies when a few proteins are available, and noise. Each of this settings require different mathematical approaches to describe network dynamis; the mathematical specification of each model is provided as Table 2.

^†^Rate of gene-switching among active and inactive states.

^‡^Abundance of the number of copies of the transcription factor *Y*.

^*^Any of these approaches is coupled with the bounded noise model^61^.

**Table 2:** Mathematical models.

Gene model			Protein model		
**Small amount of proteins and slow gene switching (Markov process)**					
	*Effect*	*Rate equation*		*Effect*	*Rate equation*
(deactiv.)	*G* → *G* − 1	*a*_1_(**z**, *t*) = *b*_0_(*t*)*G*	(transcr.)	*Y* → *Y* + 1	*a*_4_(**z**, *t*) = *sN_A_VG*
(activ.)	*G* → *G* + 1	*a*_2_(**z**, ·) = *c*_0_[*n* − *G*]	(degr.)	*Y* → *Y* − 1	*a*_5_(**z**, *t*) = *dY*
(feed.)	*G* → *G* + 1	*a*_3_(**z**, ·) = (*c*_2_*Y*^2^[*n* − *G*])/((*N*_*A*_*V*)^2^)			
**Small amount of proteins and quick gene switching (continuous equation with a Markov process)**					
		*mean-field eq.*			
		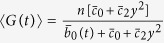	(transcr.)	*Y* → *Y* + 1	*a*_4_(*z*, *t*) = *sN_A_V*〈*G*(*t*)〉
			(degr.)	*Y* → *Y* − 1	*a*_5_(*z*, ·) = *dY*
**High amount of proteins and slow gene switching (differential equation with a Markov process)**					
(deactiv.)	*G* → *G* − 1	*a*_1_(**z**, *t*) = *b*_0_(*t*)*G*	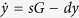		
(activ.)	*G* → *G* + 1	*a*_2_(**z**, ·) = *c*_0_[*n* − *G*]			
(feed.)	*G* → *G* + 1	*a*_3_(**z**, *t*) = *c*_2_*y*_2_(*t*)[*n* − *G*]			
**High amount of proteins and quick gene switching (coupled differential equations)**					
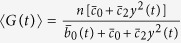			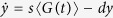		
**Parameters**					
**Smolen-Baxter-Byrne**^64^		*conversion*			
*d* = 1 *min*^−1^	*R*_*b*_ = 0.4 *min*^−1^	*ns* = *R*_*b*_ + *K*_*f*_	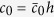		
*K*_*d*_ = 10 *nM*^2^	*K*_*f*_ = 6 *min*^−1^	*b*_0_ = *c*_0_*K*_*f*_ /*R*_*b*_	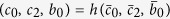		
		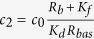	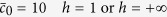		

Specification of each model as of Table 1. For every Markov process we write it in the usual birth-death notation, and write explicitly the rate function triggering any of its jumps. Every differential equation deriving from a Steady-State Hypotheses is written explicitly–for the case of genes 〈*G*(*t*)〉 denotes the average number of active genes. The system state is generally denoted as variable **z**, see Methods. Parameters are reported with comparison to the ones used by Smolen-Baxter-Byrne^64^,^66^,^67^,^68^, which we use as a base to set our parameter values. The utility parameter *h* is a scale parameter (see also^5^,^9^) set to *h* = 1 for slow gene switching and to *h* ≫ 1 for fast gene switching. The value 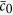 has been set to 10 so that for slow gene switching (i.e. *h* = 1) it is *c*_0_ = 10, in line with the reference work by Jaruszewicz, Zuk and Lipniacki^9^.
