# Supplementary material for: Gene switching rate determines response to extrinsic perturbations in the self-activation transcriptional network motif
Source: Sci Rep. 2016 Jun 3;6:26980. doi: 10.1038/srep26980 (PMC4891709; doi:10.1038/srep26980)
Supplement: Supplementary Information [file srep26980-s1.pdf]

Gene switching rate determines response to extrinsic perturbations in the self-activation transcriptional network motif

Supplementary Material

S. de Franciscis, G. Caravagna, G. Mauri and A. d'Onofrio

Contents

1 Mathematical and computational background **2**
1.1 Bounded noises 2
1.2 Gillespie's algorithm for systems perturbed by bounded noises 2

2 Biological details of the adopted model **3**

3 Detailed analyses **4**
3.1 Analytical results: fast gene-switching and large protein counts 4
3.1.1 Time-varying Waddington potential 6
3.2 Sensitivity analysis on protein transcription 6
3.3 Multiple noises on gene switching at once: nullification effects 7
3.4 From continuous to discrete molecular counts 8
3.5 Extended comparison with related works 8
3.6 Bounded versus unbounded forms of noise 11

List of Figures

S1 Stationary distributions of the Cai-Lin and sine-Wiener noises 3
S2 Multiple extrinsic noises affecting gene dynamics 8
S3 Comparison of models prediction for different cellular volumes 9
S4 Bounded and unbounde noises in this model 12

1 Mathematical and computational background

1.1 Bounded noises

Temporal bounded noises can be synthesized either via *stochastic differential equations*, e.g. [WT04, CW04], or via application of a bounded function to a random process, e.g. [BC05, Dim88].

We recall the definition and some basic properties of the two noises that are used in our applications, further details are available in [d'O13].

- **Cai-Lin bounded noise.** Consider the following *Langevin equation*

$$\dot{\xi}(t) = -\frac{1}{\tau}\xi(t) + \sqrt{\frac{B^2-\xi^2}{\tau(1+z)}}\eta(t), \tag{1}$$

  where $\eta(t)$ is a Gaussian white noise. As it is easy to verify, if $\xi(0) \in [-B, +B]$ then $\xi(t) \in [-B, +B]$. Moreover, it has zero mean and the same stationary autocorrelation of the Ornstein-Uhlenbeck process. The steady-state probability density of this bounded stochastic process is

$$\mathcal{P}_{st}(\xi) = C(B^2 - \xi^2)^z.$$

  The above stationary probability density exhibits different shapes according to the values of the parameter z. For $z > 0$ it is unimodal and centered in 0, while for $-1 < z < 0$ it is bimodal with two vertical asymptotes at $\xi \to \pm B$ (i.e., it is "horned").

- **Sine-Wiener bounded noise.** This is obtained by applying the bounded function $B\sin(.)$ to a Wiener process $W(t)$ yielding

$$\xi(t) = B\sin\left(\sqrt{\frac{2}{\tau}}W(t)\right). \tag{2}$$

  The noise steady-state distribution is horned and equal to the one of the Cai-Lin case for $z = -0.5$. In Figure S1 some typical steady-state distributions from Cai-Lin and sine-Wiener approaches are depicted.

Noise autocorrelation. Regardless the type of noise considered, a fundamental concept its autocorrelation, also known as serial correlation, namely the cross-correlation of a signal (a temporal series) with itself. Informally, it is the similarity between observations as a function of the time-lag between them.

Let suppose the noise has at time $\hat{t}$ a value $\xi(\hat{t})$. Autocorrelation characteristic time τ measures the time window in which the noise has a tendency to "remember" its past history, so up to $\hat{t}+\tau$ the value of $\xi(t)$ is somewhat similar to $\xi(\hat{t})$. When the noise is totally uncorrelated, like is the case of Gaussian white noise, the value $\xi(\hat{t}+dt)$ is totally independent from $\xi(\hat{t})$ for any dt (in other words one can say that $\tau \to 0$).

1.2 Gillespie's algorithm for systems perturbed by bounded noises

The simulation of a set of chemical reactions described by a stoichiometry vector ν_i and with *propensity function* $a_j(\cdot)$ is performed by using Gillespie's algorithm [Gil77], here adapted to support time-inhomogenous propensity functions arising either from the presence of noise or from the approximation discussed in the Main Text. A detailed background on such extension can be found, e.g., in [CMd13]. The translation of a birth-death process to the algebraic representation is straightforward; see [Gil77].

When the system state at time t is $\mathbf{x}_t = [x_{\text{gene}} \quad x_{\text{prot}} \quad x_\xi]^\top$, where x_{gene} is the value of G (number of active genes), x_{prot} is the number of/density of y/Y (number of proteins), and x_ξ is the noise scalar value

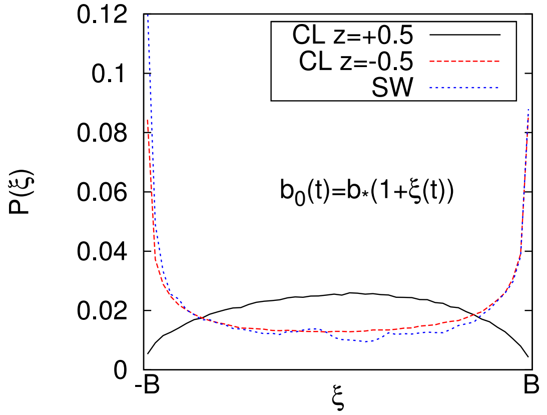

Figure S1. Stationary distributions of the Cai-Lin (horn-shaped) and the sine-Wiener (bell-alike) bounded noises suggest different types of perturbations, according to the parameters.

(e.g., such as one of those described in §1.1), the probability density function for the time of the next jump follows

$$p(\tau \mid \mathbf{x}_0, t_0) = a_0(t+\tau) \exp\left(-\int_0^{\tau} a_0(t+w)dw\right)$$

for an initial state $\mathbf{x}_0$ at time t_0, with $a_0(t+w) = \sum_j a_j(\mathbf{x}_{t+w})$. Notice that these propensity function are computed in the state that the system will be, at times afterwards t, e.g., $\mathbf{x}_{t+w}$, which will be generally different from $\mathbf{x}_t$ as the system is not homogenous in time. Given τ, the event of firing reaction j has density

$$p(j \mid \tau, \mathbf{x}_0, t_0) = \frac{a_j(t+\tau)}{a_0(t+\tau)}\,.$$

When noise is present, or when the system is hybrid – Models 2 and 3 – an evolution equation for noise/proteins (Table 2, Main Text) is coupled to common strategies to samples from such distributions, as described for instance in [CMd13].

The original Gillespie approach has scalability issues when x_{prot} is high. In particular, the total exit rate of the process is dominated by `feedback` and `degradation`, i.e.

$$a_0(t) \propto a_{\text{feed}}(t) + a_{\text{degr}}(t)$$

which grows as $\mathcal{O}(x_{\text{prot}}^2)$ when x_{prot} is high, as $x_{\text{gene}} < 2$. If we assume noise to change smoothly, we can consider the propensities to be time-homogenous and observe that

$$\langle \tau \rangle = a_0(t)^{-1}\,.$$

Hence the average time-increment of each jump is small (precisely, decreases quadratically with the number of proteins), and it might get computationally hard to sample many independent trajectories of the system, unless we switch to an hybrid representation of x_{prot}. In this case, x_{prot} varies via a differential equation, the total contribution of the rates to $a_0(t)$ is smaller so allowing the system to leap over longer jumps, with the overhead of integrating such a differential equation.

2 Biological details of the adopted model

The simple model that we adopted, introduced in literature by Smolen, Baxter and Byrne in their well-known paper [SBB98], represents a single transcription factor (TF, variable Y) that self-activates. In

detail:

1. the gene producing the TF has only one promoter;
2. the active form of the TF is its dimer;
3. the dimerization rate is very fast, thus the dimer is at equilibrium with the TF and the dimerization process is not directly represented in the model;
4. the activation of the gene is thus triggered by its binding with the dimer;
5. however, also in absence of binding there is a small baseline activation rate, independent from the gene binding to dimers.

Besides the assumptions made in [SBB98], we further assume that:

6. due to the interplay with unknown signals (modelled as bounded noise) the deactivation rate stochastically fluctuates.

The mathematical translation of this biological scenario leads to a stochastic model with total activation rate in bimolecular form

$$\mathsf{act}(Y) = c_0 + c_2 Y(Y-1)$$

which, according to the the relation between reaction rate equations and propensity functions for stochastic systems – see, e.g., [Gil77] – leads to the approximation

$$\overline{\mathsf{act}}(Y) = c_0 + c_2 Y^2$$

in the case of large amount of the TF as

$$\mathsf{act}(Y) \approx c_0 + c_2 Y^2 = \overline{\mathsf{act}}(Y).$$

We used this form of the rate in all settings, as stochastic models where the dimerization follows law of the type Y^2 instead of $Y(Y-1)$ are widely used in the literature [BG05, WSW16].

3 Detailed analyses

In this section, we drop the bar over the symbols $\bar{c}_0$, $\bar{c}_2$ and $\bar{b}_0$. This is an abuse with respect to the main text, but it is done for the sake of the simplicity of notation.

3.1 Analytical results: fast gene-switching and large protein counts

Concerning model D (fast gene switching and large protein counts), we may give some analytical results of interest. Here we assume that the parameters of the FC model are such that the system is multistable. We define the utility functions $F(U)$ (with $U \geq b_l$), $G(U)$ (with $U \in [b_l, b_r]$) and $H(u)$ (with $U \leq b_r$) which compute, respectively, the smallest, the intermediate and the largest of the three real solutions of the equation:

$$sn \frac{c_0 + c_2 y^2}{U + c_0 + c_2 y^2} - dy = 0.$$

First suppose that $b_*(1-B) \geq b_l$. Thus, if $y(0) \in [0, G(b_*(1-B))]$ then for large times $y(t) \in [F(b_*(1+B)), F(b_*(1-B))]$. This results follows from the following differential inequalities:

$$sn \frac{c_0 + c_2 y^2}{b_*(1+B) + c_0 + c_2 y^2} - dy \leq y' \leq sn \frac{c_0 + c_2 y^2}{b_*(1-B) + c_0 + c_2 y^2} - dy \tag{3}$$

Indeed, the above inequalities imply that $y_a(t) < y(t) < y_b(t)$ where $y_a(t)$ solve the following ODEs:

$$y_a' = sn\frac{c_0 + c_2 y_a^2}{b_*(1+B) + c_0 + c_2 y_a^2} - dy_a,\ y_a(0) = y(0)$$

$$y_b' = sn\frac{c_0 + c_2 y_b^2}{b_*(1-B) + c_0 + c_2 y_b^2} - dy_b,\ y_b(0) = y(0).$$

As it is easy to verify, these ODEs are such that for large times $y_a \geq F(b_*(1+B))$ and $y_b \leq F(b_*(1-B))]$. Similarly, if $b_*(1+B) \geq b_r$ and $y(0) \geq G(b_*(1+B))$ then for large times it must be $y(t) \in (H(b_*(1-B)), H(b_*(1+B)))$. Moreover, if

$$b_*(1-B) < b_l < b_* < b_*(1+B) < b_r$$

and $y(0) < F(b_l)$ it apparently follows the quite neutral result that for large times it must be $y(t) \in [H(b_*(1-B)), F(b_*(1+B))]$. However, if in a time instant $\widehat{t}$ it is $y(\widehat{t}) \geq G(b_*(1+B))$ then (based on the above inequalities) it must be that for $t > \widehat{t}$ it is $y(t) \in [H(b_*(1-B)), G(b_*(1+B))]$. Due to the random nature of the perturbations, the existence of such a $\widehat{t}$ is very likely, if not sure. Indicating with

$$\mathcal{P}_L(Y,t) = \mathcal{P}\Big(y(t) \in (Y, Y+dY)\Big| y(0) \in [0, F(b_l)]\Big)$$

and with $\mathcal{P}_L^{st}(Y) = \mathcal{P}_L(Y, +\infty)$ the above results suggest that in the case $y(0) \in [0, F(b_l)]$:

- if $b_* \in (b_l, b_r)$ and $b_*(1-B) > b_l$ then $\mathcal{P}_L^{st}(Y)$ it is null outside $[F(b_*(1+B)), F(b_*(1-B))]$.
- if $b_* \in (b_l, b_r)$, $b_*(1-B) < b_l$, and $b_*(1+B) > b_r$ then $\mathcal{P}_L^{st}(Y)$ it is null outside $[H(b_*(1+B)), H(b_*(1-B))]$

The second result suggests that defining the "order parameter" $\langle y \rangle$, and considering its variation with $B \geq 0$, it may undergo a first order transition at $b_*(1-B) = b_l$, because there $\langle y \rangle(B)$ is discontinuous. A similar result can be obtained with reference to the upper branch of the bifurcation diagram. Indeed, defining

$$\mathcal{P}_H(Y,t) = \mathcal{P}\Big(y(t) \in (Y, Y+dY)\Big| y(0) \geq F(b_r)\Big)$$

and $\mathcal{P}_H^{st}(Y) = \mathcal{P}_H(Y, +\infty)$ it follows that in the case $y(0) \geq F(b_r)$

- if $b_* \in (b_l, b_r)$ and $b_*(1+B) > b_r$ then $\mathcal{P}_H^{st}(Y)$ it is null outside $[H(b_*(1+B)), H(b_*(1-B))]$.
- if $b_* \in (b_l, b_r)$, $b_*(1+B) > b_r$, and $b_*(1-B) > b_l$ then $\mathcal{P}_H^{st}(Y)$ it is null outside $[F(b_*(1+B)), F(b_*(1-B))]$

With respect to the unconditioned probability density $\mathcal{P}(Y,t)$ the above results show that its asymptotic behavior (in the functional space of the probability densities) strongly depends on its initial conditions. In other words, for small to moderate amplitude B of the noise $y(t)$ has two distinct stochastic attractors (two distinct phases). By increasing B over a threshold B_C the asymptotic behavours changes and there is a unique attractor. Of course, if the nosie amlitude is slightly over the threshold and then decrase to a value under the threshold, one passes for a scenario with a unique attractors to another one with two attractors.

For example, in theory, in numerical simulations with $y(0) \in [0, F(b_l)]$ we would expect to observe such a first order transition, i.e. that it exists a B_c (with $b_*(1-B_c) = b_l$) such that for $0 \leq B < B_c$ the stationary density $\mathcal{P}_L^{st}(Y)$ is unimodal and located at low values of y, whereas for $B_c < B$ the density jumps at larger values of y.

As far as this first-order transition due to the double distinct attractors of $y(t)$ that disappear when the parameter B increases and crosses a threshold, we recall that the non-uniqueness of the asymptotic attractors in a physical system (and, as a consequence, in its associated Fokker-Planck equation) is a landmark of genuine phase transitions, as discussed in depth by Shiino [Shi87] (see also [VdBPT94]). This phenomenon is rarely observed in non-spatial systems, and it makes our observed first-order transition quite close to 'true' phase transitions.

We note that in the statistical physics the search for multiple attractors has traditionally been done in non-linear Fokker-Planck equations, obtained by applying mean field approximations to large number of interacting nonlinear systems (e.g. by diffusion [Shi87, VdBPT94]). In our case, instead, the system is unique and non-spatial, and the Fokker-Planck equation (describing the jont properties of the noise and of the variable y) is linear, although it has a different peculiar characteristic: it has degenerate diffusion because the dynamics of y does not depend on white noises.

Finally, if the noise amplitude B is further increased to values $B > B_d$, where $b : m(1 + B_d) = b_r$, we expect that $\mathcal{P}_L^{st}(Y)$ gets bimodal and $\langle y \rangle(B)$ decreases. Summarizing, apart the a fist-order transition at $B = B_C$, at $B = B_d$ there is a noise-induced transition from unimodality to bimodality (and a smooth decrease of $\langle y \rangle(B)$). Two similar transitions should, thus, also be expected in the case $y(0) > F(b_r)$.

3.1.1 Time-varying Waddington potential

Recently Verd et al. [VCJ14] introduced in the framework of Systems Biology models affected by *deterministic* time-varying perturbations the concept of time-varying Waddington's potential. We believe that this potential might be extremely useful also for stochastically perturbed systems like ours. Namely, to our model:

$$y' = s\frac{n(c_0 + c_2 y^2)}{b_0(t) + c_0 + c_2 y^2} - dy,$$

it is associated the following time-varying potential:

$$W(y,t) = \frac{d}{2}y^2 - sn\left(y - \frac{b_0(t)}{\sqrt{b_0(t) + c_0}}\frac{1}{\sqrt{c_2}}Arctan\left(y\sqrt{\frac{c_2}{b_0(t) + c_0}}\right)\right)$$

The shape of the potential $W(x,t)$, and the number and basin of attraction of its 'potential holes', stochastically change in the time. Thus, for example, in case of small to moderate fluctuations of $b_0(t)$ the corresponding irreversible transition 'low to large' ('large to low') values of y can heuristically be read as the irreversible 'capture' by large (low) value attractors of a trajectory initially confined in a potential hole centered at low values of y.

3.2 Sensitivity analysis on protein transcription

When the continuous model is in it multi-stability region for non-degenerate s results shown in the Main Text are unchanged. Consider in fact the noise-free continuous equations in model D (Table 2, Main Text), replace $\langle G(t) \rangle$ in the differential equation for proteins.

The equilibrium values for proteins are the zeros of the equation $\dot{y} = 0$, which reads as

$$s\frac{n\left[c_0 + c_2 y^2\right]}{b_0 + c_0 + c_2 y^2} - dy = 0$$

for the noise-free system (i.e., the network isolated from the external environment). This polynomial is equivalent to

$$y^3 dc_2 - y^2 snc_2 + y(db_0 + dc_0) - snc_0 = 0$$

when $y^2 \neq -(b_0 + c_0)/c_2$ and has at most 3 solutions which can be easily retrieved by applying the cubic formula for root finding.

For a general polynomial we can compute exactly its roots; by setting $\alpha = dc_2$, $\beta = -nc_2$, $\gamma = db_0 + dc_0$ and $\delta = -nc_0$ we have

$$p(y, s) = y^3 \cdot \alpha - y^2 \cdot \beta \cdot s + y \cdot \gamma - s \cdot \delta = 0$$

whose discriminant is

$$\begin{aligned}\Delta(s) &= 18\alpha\beta s\gamma\delta s - 4(\beta s)^3\delta s + (\beta s)^2\gamma^2 - \alpha\gamma^3 - 27\alpha^2(\delta s)^2 \\ &= 18\alpha\beta\gamma\delta s^2 - 4\beta^3\delta s^4 + \beta^2\gamma^2 s^2 - \alpha\gamma^3 - 27\alpha^2\delta^2 s^2 \\ &= 4\beta^3\delta s^4 + (18\alpha\beta\gamma\delta + \beta^2\gamma^2 + 27\alpha^2\delta^2)s^2 - \alpha\gamma^3 .\end{aligned}$$

When $\Delta(s) > 0$ then $p(y, s)$ has 3 roots; numerical conditions for this to hold are

$$\Delta(s) > 0 \iff 2.2255 \leq s \leq 143.7882 .$$

This means that for any $s \in [2.2255, 143.7882]$ we can find conditions to have an hysteresis plot equilibria such as the one in Figure 1. Such plots will only differ for the values of the equilibrium values $(y_{\text{low}}, y_{\text{high}})$ and (b_l, b_r), as a function of s.

Notice that we picked – on purpose – a set of parameters values in which all the 3 roots exists, as this is the interesting case where multi-stability can emerge. Thus, for any of such plots, we can always retrieve our conclusions when the noise baseline value (b_0) is close enough to b_l - which is the situation described in the Main Text. For this reason, we did not include any quantitative statement on our predictions (like specific protein counts) being much aware on the dependency on the specific value of s.

3.3 Multiple noises on gene switching at once: nullification effects

We show that, if two independent noise sources are applied to both the process of gene switching between inactive and active states and viceversa the extrinsic noise effect is averaged out.

Let us consider the continuous case, which is model D of our manuscript; the equation for the average number of active genes can be extended to account for a noise in the activation baseline rate, c_0 in the main text, yielding

$$\langle G(t) \rangle_{\text{new}} = \frac{n \left[c_0(t) + c_2 y^2(t)\right]}{b_0(t) + c_0(t) + c_2 y^2(t)}$$

Let us consider the two noises separately. First, applying the bounded noise in b_0, we would have that $\langle b_0(t) \rangle = b_0$ and that, for any t, $b_0(t) \in [b_0(1 - \hat{B}), b_0(1 + \hat{B})]$ which would lead, case of the strongest possible noise intensity ($\hat{B} = 1$) to $b_0(t) \in [0, 2b_0]$. In this case the state equation for gene number fluctuates between:

$$\langle G \rangle^{b0}_{\min} = \frac{n \left[c_0 + c_2 y^2(t)\right]}{2b_0 + c_0 + c_2 y^2(t)} \qquad \langle G \rangle^{b0}_{\text{Max}} = \frac{n \left[c_0 + c_2 y^2(t)\right]}{c_0 + c_2 y^2(t)} = n. \tag{4}$$

With our parameter setting, i.e. $c_0 = 10$ $c_2 = 16$ $b_0 = 150$, we will obtain for a low level of protein equilibrium $y^{eq}_{low} \simeq 1$, $\langle G \rangle^{b0}_{\min} \simeq \frac{n}{9}$, thus the magnitude of fluctuations is $\Delta G^{b0}_{low} \simeq n8/9$. On the other hand for high level of protein equilibrium $y^{eq}_{high} \simeq 4$, $\langle G \rangle^{b0}_{\min} \simeq \frac{n}{2.12}$, thus the magnitude of fluctuation now is $\Delta G^{b0}_{high} \simeq n/2$.

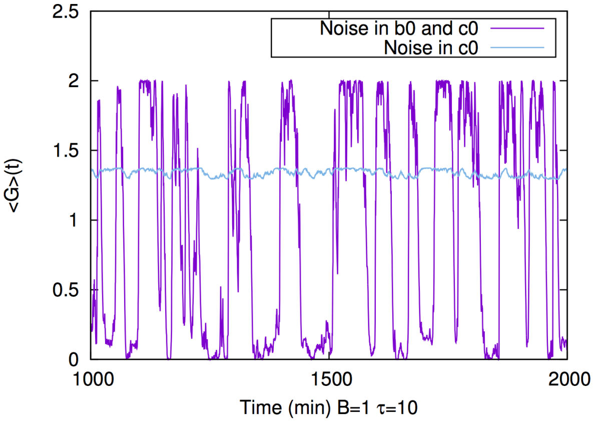

Figure S2. Multiple extrinsic noises affecting gene dynamics. Here we compare the situation of and extrinsic bounded noise affecting only deactivation ($b_0(t)$) and both activation and deactivation ($b_0(t)$ and $c_0(t)$) of G, in the very extreme case of maximum noise strength ($B = 1$). This shows how the magnitude of fluctuations in the average number of active genes is almost nullified in the latter case, compared to the former.

When the noise is applied to c_0, the state equation for gene number fluctuates between:

$$\langle G \rangle^{c0}_{\min} = \frac{nc_2 y^2(t)}{b_0 + c_2 y^2(t)} \qquad \langle G \rangle^{b0}_{\mathrm{Max}} = \frac{n\left[2c_0 + c_2 y^2(t)\right]}{b_0 + 2c_0 + c_2 y^2(t)}. \tag{5}$$

The same consideration of the former case lead us, for y^{eq}_{low} to $\langle G \rangle^{c0}_{\min} \simeq \frac{n}{10}$ and $\langle G \rangle^{c0}_{\mathrm{Max}} \simeq \frac{n}{4}$, and the magnitude of fluctuations is $\Delta G^{c0}_{low} \simeq n\frac{3}{20} < \Delta G^{b0}_{low}$. For y^{eq}_{high}, $\langle G \rangle^{c0}_{\min} \simeq \frac{n}{1.6}$, $\langle G \rangle^{c0}_{\mathrm{Max}} \simeq \frac{n}{1.54}$, and the magnitude of fluctuations $\Delta G^{c0}_{high} \simeq 0 < \Delta G^{b0}_{high}$.

For further clarification, we plot $\langle G(t) \rangle$ in the case of the noise source affecting both activation and inactivation of G; see Figure S2. The same conclusions can be drawn when the same noise source is considered.

3.4 From continuous to discrete molecular counts

The discretization of a model do not necessarily preserve the properties of its continuous counterpart. Different volumes – and hence different normalization constants, Fig. 7 (Main Text), and discretization – imply different reference biological systems. In general, to the best of our knowledge, it is not possible to automatically derive results on discrete-level of proteins/genes from a phase-diagram. In practical terms, this means that there is no theoretical guarantee that standard analysis on the continuous version of the model (model D) can shed any light on the network functioning in different settings (models A,B and C). This is indeed what we observe for this system, see Figure S3.

3.5 Extended comparison with related works

In this section we comment on three models of the self-activation transcriptional network motif and its relation with extrinsic Gaussian noises. In two cases this is the only form of stochasticity considered [LaLGLL09, LJ04], in a unique case this is in combination with intrinsic fluctuations but without gene-switching noise [ARLSG13].

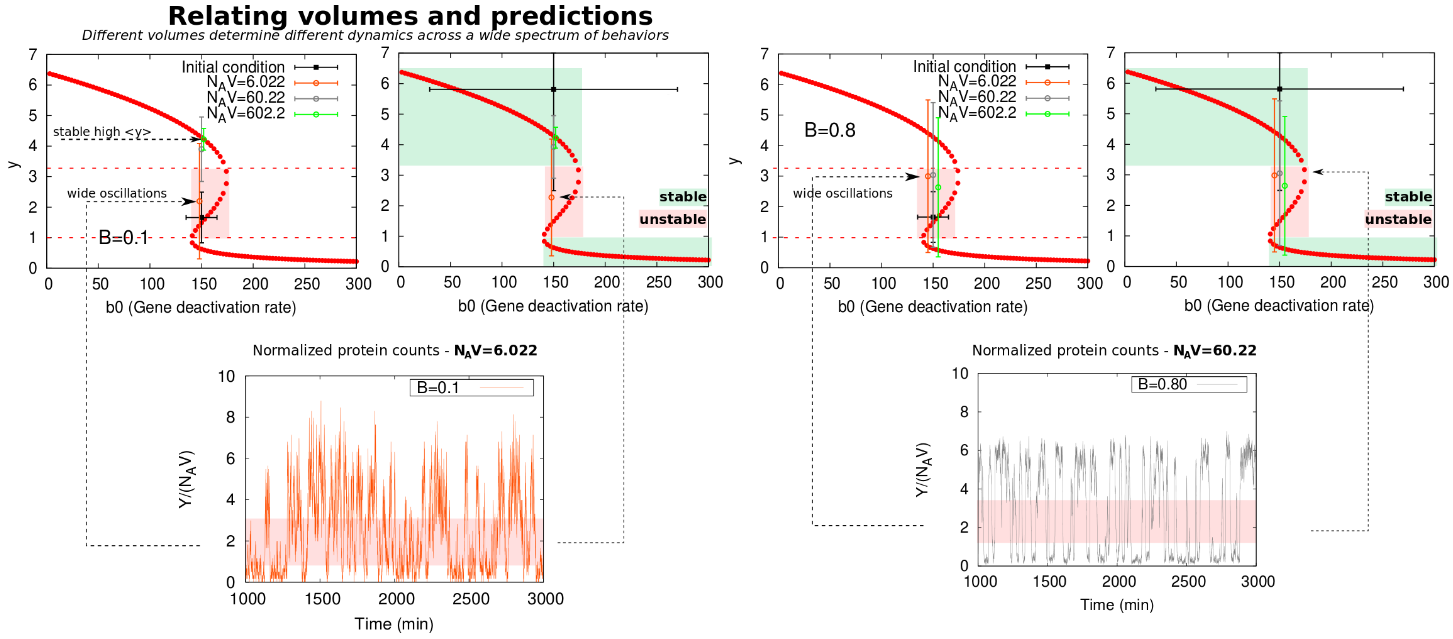

Figure S3. In these plots, we project the hysteresis diagram for the noise-free fully continuous model – Fig 2a of the Main Text – along with different initial conditions (as of y_0 and b_0) of the analogous discrete model (conditions vary in the left and right plots). Top and bottom panels differ for noise intensity B. Possible trajectories are pictured with their spanned values as bars. We can observe that the predicted behaviors are independent of the initial condition, but rather dependent on the cellular volume (via normalization $N_A V$). Notice that for low noise, $B = 0.1$, oscillations are only predicted for low-volume cells, $N_A V = 6.02$. While for higher noise $B = 0.1$ no matter what the volume is the dynamics oscillates across high and low protein counts.

As we mentioned in the main text, this model in absence of extrinsic noise reads as follows:

$$y' = s\frac{n(c_0 + c_2y^2)}{(c_0 + c_2y^2) + b_0} - dy,$$

but, in the literature, e.g., [SBB98, LJ04, LaLGLL09, ARLSG13], it is often written in the algebraically equivalent form

$$\dot{y} = R_b + \frac{K_f y^2}{K_d + y^2} - dy, \tag{6}$$

where R_b and the sum $K_f + R_b$ can be, respectively, legitimately read as a baseline and an "asymptotic" production rates. However, since it holds that

$$R_b = ns\frac{c_0}{c_0 + b_0}$$

$$K_f = ns$$

$$K_d = \frac{c_0 + b_0}{c_2}$$

it follows that the parameters R_b, K_f and K_d must not be dealt with as they were independent. In particular, a fluctuation of the baseline rate R_b cannot be deconvolved by fluctuations in the other two parameters.

Unfortunately this is what happens in literature. For example, in [LaLGLL09] the Smolen-Baxter-Byrne model was studied in the framework of the above mentioned continuous approximation with fast gene-switching and large number of proteins. The authors of [LaLGLL09] analytically investigated the consequences of white stochastic oscillations affecting the baseline production rate K_d and/or the parameter K_f. Thus, the model in [LaLGLL09] reads as follows

$$\dot{y} = R_b(1 + \xi_0(t)) + K_f(1 + \xi_1(t))\frac{y^2}{K_d + y^2} - dy\,. \tag{7}$$

Unfortunately, according to what we above showed, the stochastic differential equation (7) does not correspond to a biologically meaningful scenario, unless both $\xi_0(t) = \xi_1(t)$ and, of course, the noises are bounded. We note here that, in such a particular case, eq. [LaLGLL09] can be read as a model of fluctuations in the parameter s.

In [LJ04] it is investigated a model where both the baseline protein production rate and the degradation rate were perturbed by white noises, yielding the following stochastic differential equation

$$\dot{y} = R_b(1 + \xi_0(t)) + K_f\frac{y^2}{K_d + y^2} - d(1 + \xi_d(t))y\,, \tag{8}$$

Again, isolated oscillations of the parameter R_b are not meaningful.

As briefly mentioned in the introduction, by means of semi-analytical methods, joining the Wentzel-Kramers-Brillouin (WKB) approximation and numerical simulations, Assaf and colleagues recently investigated the interplay between extrinsic and intrinsic noise in the circuit of a self-transcription factor with a sharp positive feedback [ARLSG13]. The biological differences between their model and ours are worth to be described in some detail.

Indeed, in their main model the extrinsic noise perturbs the production rate of the transcription factor, and the perturbation is state-dependent being active only if the state of the protein is "high". Indeed, under the implicit hypothesis that the gene switching velocity is large they assume the following probability law for the production of the transcription factor in the time interval $(t, t + dt)$

$$\mathcal{P}(Y \to Y + 1) = dt\; A(\alpha_0 + (1 - \alpha_0 + \xi(t))\theta(Y - Y_*)) \tag{9}$$

where $\theta(.)$ is the Heavyside function and $\alpha_0 \ll 1$. Thus if $Y(t)$ is under the threshold then the production rate of the transcription factor is unperturbed.

The mechanism through which this state-dependent fluctuation of the production rate can be enacted is not specified. In absence of such specification, the asymmetry of the perturbation acting on the baseline and on the large protein synthesis rates remains unclear.

Note that in [ARLSG13] it is also briefly investigated a model where a smooth feedback is enacted (as in the Smolen-Baxter-Byrne model [SBB98, SBB99]):

$$\mathcal{P}(Y \to Y + 1) = dt\; A\left(\alpha_0 + (1 - \alpha_0 + \xi(t))\frac{Y^2}{K_d + Y^2}\right) \tag{10}$$

Thus, the perturbation adopted in [ARLSG13] for such a smooth case is equivalent to the perturbation of the parameter K_f alone investigated in the paper [LaLGLL09] in the case $\xi_0(t) = 0$.

We note here that this equivalence can be extended to the 'sharp' model defined by 9 because in absence of extrinsic noise eq 9 is the limit case of a generalization of our model B (fast gene switching and small number of proteins) with constant b_0. Indeed, in the hypothesis that gene switching is fast and that the activation is caused by 'Q-meric' forms (and not dimeric), proceeding as in our main text one gets (in absence of noise on b_0):

$$\mathcal{P}(Y \to Y + 1) = dtsN_AV\langle G(t)\rangle = dtsN_AV\frac{n\left[c_0 + c_2y^Q\right]}{b_0 + c_0 + c_2y^Q}, \tag{11}$$

which may be rewritten as follows:

$$\mathcal{P}(Y \to Y+1) = dtA(\alpha_0 + (1-\alpha_0)\frac{(Y/Y^*)^Q}{1+(Y/Y^*)^Q}, \tag{12}$$

where

$$Y_* = (\frac{b_0 + c_0}{c_2})^{1/Q}$$

$$A\alpha_0 \approx sN_A V n \frac{c_0}{b_0 + c_0}.$$

Thus, if Q is sufficiently large eq. (12) reads:

$$\mathcal{P}(Y \to Y+1) \approx dt\; A\,(\alpha_0 + (1-\alpha_0)\theta(Y - Y_*))$$

Summarizing, we may say that for both the sharp and the smooth models of [ARLSG13] the extrinsic noise acts in a biologically unrealistic way, equal (for $Q = 2$) or remindful (for $Q \gg 1$) of that investigated in [LaLGLL09] in the case $\xi_0(t) = 0$.

As far as the type of extrinsic noise is concerned, Assaf and coworkers considered an unbounded Orenstein-Uhlenbeck noise defined as follows

$$\dot{\xi} = -\frac{1}{\tau}\xi + \sqrt{\frac{2\sigma_{ex}}{\tau}}\eta(t)\,,$$

where $\eta(t)$ is a unitary white noise. In this way the stationary probability density function of the noise ξ is Gaussian with variance σ_{ex}. However, from the above equations it follows that it must be

$$\xi(t) > 1 - \alpha_0\,.$$

One might roughly consider tolerable the error if

$$2\sigma_{ex} < 1 - \alpha_0\,,$$

which, however, does not seem the case in [ARLSG13]. Indeed in the simulations presented in [ARLSG13] the employed "stochastic bifurcation parameter" is the ratio between the standard deviation of the Orenstein-Uhlenbeck noise and a parameter named μ. This parameter is defined as the average of the (transitory) "quasi-stationary distribution about the high state" [ARLSG13]. Since in their simulations the high state is large (it ranges from 300 to 5000), even μ has to be large. Indeed, we simulated the sharp feedback model by means of the (exact) Gillespie algorithm and we got that the average value of the probability density function is very close to the high equilibrium state. This means that the standard deviation σ_{ex} in most of the simulations reported in [ARLSG13] largely exceeds $0.5(1 - \alpha_0)$.

3.6 Bounded versus unbounded forms of noise

Consider the continuous version of the model and the phase-transition that we observe. The fact that more common approaches based on unbounded extrinsic perturbations will not predict such dynamics is one of the motivations for our work. It is indeed the case that, for the parameters that we have considered, the network interplaying with unbounded white/colored noises predicts oscillations among high and low levels of protein Y.

We picture evidences of this from single simulations of the system under the effect of one of the most famous models of unbounded noise, see Figure S4. We there show two single simulations of the system under the effect of an Ornstein-Uhlenbeck noise ξ generated from the following stochastic differential equation

$$d\xi = -\frac{\xi}{\tau}\,dt + \sigma\,dW_t$$

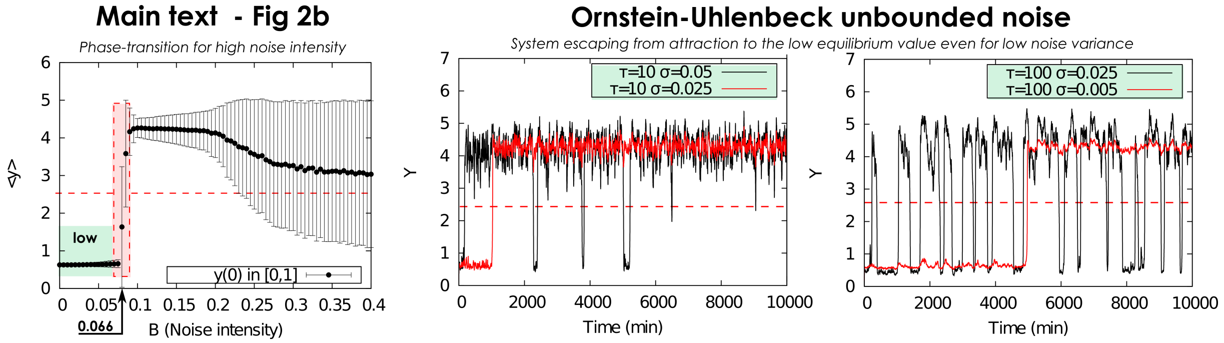

Figure S4. Comparison of models prediction with bounded and unbounded noise. On the left, a figure from the Main Text shows a phase transition (for values of noise intensity $B > B_*$, with $B_* \approx 0.066$ and $\tau = 10$) from low to high values of $\langle y \rangle$. On the right, two simulations of the system under the effect of an Ornstein-Uhlenbeck.

where we "compare" the Ornstein-Uhlenbeck noise variance (σ) to the intensity of the bounded noise. In support of our conclusions we cal also observe that, even for $\sigma \ll B_*$ the system is predicted to stabilize to either its higher protein state, or to oscillate among equilibria - as a function of τ. We note that this happens with non-zero probability for all parameter settings of such noises, by the infinite support of the Gaussian distribution. As such, we feel that this kind of behaviours – emerging only with unbounded noises – could be considered as a modeling artifact.

For this reason, caution should be adopted to distinguish predictions more likely to be "realistic" (i.e., adhere to underlying physics of the system) from modelling artifacts. In this sense, we believe that the type of behaviour that we have shown, i.e., a phase-transition, shall be considered a peculiari noise-effect, as it is not whatsoever reproducible in canonical white/colored-noise modeling contexts.

References

ARLSG13. Michael Assaf, Elijah Roberts, Zaida Luthey-Schulten, and Nigel Goldenfeld. Extrinsic noise driven phenotype switching in a self-regulating gene. *Phys. Rev. Lett.*, 111:058102, Jul 2013.

BC05. R. V. Bobryk and A. Chrzeszczyk. Transitions induced by bounded noise. *Physica A: Statistical Mechanics and its Applications*, 358(2-4):263–272, 2005.

BG05. A-B Besya and A Gronlund. Intrinsic phenotypic stability of a bi-stable auto regulatory gene. *Scientific Reports*, 6:828–850, 2005.

CMd13. G. Caravagna, G. Mauri, and A. d'Onofrio. The interplay of intrinsic and extrinsic bounded noises in biomolecular networks. *PLoS ONE*, 8(2):e51174, 2013.

CW04. G.Q. Cai and C. Wu. Modeling of bounded stochastic processes. *Probabilistic Engineering Mechanics*, 19(3):197 – 203, 2004.

Dim88. Mikhail Dimentberg. *Statistical dynamics of nonlinear and time-varying systems.* Research Studies Press, 1988.

d'O13. Alberto d'Onofrio. *Bounded Noises in Physics, Biology, and Engineering.* Modeling and Simulation in Science, Engineering and Technology. Springer New York, 2013.

Gil77. Daniel T Gillespie. Exact stochastic simulation of coupled chemical reactions. *The journal of physical chemistry*, 81(25):2340–2361, 1977.

LaLGLL09. Xue-Mei Liu, Hui-Zhang Xie ans Liang-Gang Liu, and Zhi-Bing Li. Effect of multiplicative and additive noise on genetic transcriptional regulatory mechanism. *Physica A*, 388:392–398, 2009.

LJ04. Quan Liu and Ya Jia. Fluctuations-induced switch in the gene transcriptional regulatory system. *Physical Review E*, 70:041907, 2004.

SBB98. Paul Smolen, Douglas A Baxter, and John H Byrne. Frequency selectivity, multistability, and oscillations emerge from models of genetic regulatory systems. *American Journal of Physiology-Cell Physiology*, 274(2):C531–C542, 1998.

SBB99. Paul Smolen, Douglas A. Baxter, and John H. Byrne. Effects of macromolecular transport and stochastic fluctuations on dynamics of genetic regulatory systems. *American Journal of Physiology - Cell Physiology*, 277(4):C777–C790, 1999.

Shi87. Masatoshi Shiino. Dynamical behavior of stochastic systems of infinitely many coupled nonlinear oscillators exhibiting phase transitions of mean-field type: H theorem on asymptotic approach to equilibrium and critical slowing down of order-parameter fluctuations. *Physical Review A*, 36(5):2393, 1987.

VCJ14. Berta Verd, Anton Crombach, and Johannes Jaeger. Classification of transient behaviours in a time-dependent toggle switch model. *BMC Systems Biology*, 8(1):1–19, 2014.

VdBPT94. C Van den Broeck, JMR Parrondo, and R Toral. Noise-induced nonequilibrium phase transition. *Physical review letters*, 73(25):3395, 1994.

WSW16. AM Walczak, M Sasai, and PG Wolynes. Self-consistent proteomic field theory of stochastic gene switches. *Biophysical Journal*, 88:22951, 2016.

WT04. Horacio S. Wio and Raúl Toral. Effect of non-gaussian noise sources in a noise-induced transition. *Physica D: Nonlinear Phenomena*, 193(1-4):161–168, 2004.
